# Reusing the Wasted Energy of Electrochromic Smart Window for Near‐Zero Energy Building

**DOI:** 10.1002/advs.202406232

**Published:** 2024-09-16

**Authors:** Yunfei Xie, Ruonan Huang, Meini Li, Ningzhi Cao, Xiaoteng Jia, Caiyun Wang, Danming Chao

**Affiliations:** ^1^ College of Chemistry Jilin University Changchun 130012 China; ^2^ State Key Laboratory of Integrated Optoelectronics College of Electronic Science and Engineering Jilin University Changchun 130012 China; ^3^ Intelligent Polymer Research Institute Faculty of Engineering and Information Sciences Innovation Campus University of Wollongong North Wollongong NSW 2500 Australia

**Keywords:** electroactive polymer, electrochromic, energy storage, smart window

## Abstract

Electrochromic smart windows (ESWs) are an effective energy‐saving technology for near‐zero energy buildings. They consume electric energy unidirectionally during a round‐trip coloring‐bleaching process, with the energy involved in the bleaching process being wasted. It is highly desirable to reuse this wasted electric energy directly and/or transfer it into other energy storage equipment, further enhancing the overall efficiency of electric energy usage. Herein, a zinc anode‐based ESW (ESW‐PZ) is reported that not only has fascinating visible–near‐infrared (VIS‐NIR) dual‐band electrochromic performance (a high optical contrast of 63%) but also showcases good energy storage characteristics (a wide voltage window of 2.6 V and a high energy density of 127.5 µWh cm^−2^). The buildings utilizing ESW‐PZ to modulate indoor environments demonstrated an average annual energy saving of 366 MJ m^−2^ based on energy simulations, which is about 16% of the total energy consumption. Impressively, a high utilization efficiency of 90% (855 mWh m^−2^) of the wasted electric energy is realized through an ingenious circuit‐switching strategy, which can be reused to power small household appliances.

## Graphical abstract

A novel electrochromic smart window with satisfactory energy‐saving behavior and energy‐reuse properties is developed. It demonstrates a 16% reduction in building energy consumption, and up to 90.0% of the wasted electric energy can be reused through a smart circuit‐switching strategy.

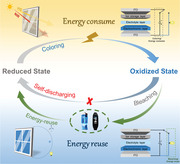

## 1 Introduction

The swift growth of the global population and the rapid urban development have placed unprecedented pressure on society due to the escalating energy demand of buildings.^[^ ^1^, ^2^, ^3^ ^]^ Currently, over 30% of the total building energy consumption is used for HVAC (heating, ventilation, and air conditioning) to maintain a comfortable indoor environment.^[^ ^4^, ^5^, ^6^ ^]^ This excessive energy consumption contributes to the emission of substantial amounts of carbon dioxide, consequently exacerbating global warming and leading to significant ramifications for human society.^[^ ^7^ ^]^ Among the various parts of a building, windows are considered the least energy‐efficient due to the inevitable heat exchange with the external environment through light and heat radiation. Therefore, enhancing the light/thermal management capabilities of windows is of great significance for minimizing energy consumption and upholding energy efficiency standards in buildings.^[^ ^8^, ^9^, ^10^ ^]^

Smart window technology has emerged to address the need for adjusting the transmittance of solar radiation, thereby reducing energy consumption associated with HVAC systems.^[^ ^11^, ^12^, ^13^, ^14^, ^15^ ^]^ Since the introduction of “smart windows” by C.M. Lampert and Granqvist, they have been attracting extensive attention due to their excellent light regulation and thermal management properties, showcasing great potential to reduce the energy consumption of buildings.^[^ ^16^, ^17^ ^]^ Among them, electrochromic smart window technology as the earliest option possesses overwhelming advantages such as accurate color control, fast color‐changing speed, and bistable characteristics. It has now been preliminarily industrialized and commercially applied.^[^ ^18^, ^19^, ^20^, ^21^ ^]^ Different types of ESWs have been increasingly manufactured and evaluated recently,^[^ ^22^, ^23^, ^24^, ^25^, ^26^, ^27^ ^]^ achieving a desirable energy‐saving performance of ≈10%.^[^ ^28^, ^29^, ^30^ ^]^ These studies demonstrate that ESWs can efficiently reduce building energy consumption.

During the operation of ESW, the electrochromic material undergoes reversible switching between bleached and colored states through redox reactions when an external potential is applied, thus enabling on‐demand light and thermal management.^[^ ^31^, ^32^, ^33^, ^34^ ^]^ Therefore, this color‐switching behavior is inevitably accompanied by energy transfer. The coloring course of electrochromic materials is attributed to an oxidation reaction, corresponding to a charging process. The electric energy involved is not consumed but instead stored in ESWs.^[^ ^35^, ^36^, ^37^, ^38^ ^]^ During the bleaching process, the stored electric energy is consumed through self‐discharging and wasted. In a word, the ESW consumes energy unidirectionally during a round‐trip coloring‐bleaching process, with the involved energy being wasted through the discharging (bleaching) process. It is imperative to reuse this wasted electric energy, thereby boosting the overall efficiency of electric energy usage. This recycled energy may be used to charge small appliances or transferred into other energy storage equipment. Recently, Li and Elezzabi proposed a Zinc‐based electrochromic energy storage device with a bifunctional of electrochromism and energy storage.^[^ ^39^, ^40^, ^41^ ^]^ It was ascribed to the configuration and the electrochemical reversibility of zinc anode. However, the amount of recovered energy was below 50%. To achieve a high energy recovery efficiency along with excellent electrochromic properties, the electroactive material must possess high energy storage performance in addition to the merits of simultaneous excellent electrochromic and good optical/electrical bistability. Moreover, a rational circuit design is required for this innovative electrochromic system featuring energy‐reuse with high efficiency.

Herein, we synthesized an electroactive polyamide bearing densely distributed oligoaniline groups. This material exhibited good processability, dual‐band absorption in the visible to near‐infrared (VIS‐NIR) spectra, and reversible zinc ion doping/dedoping ability. When coupled with a zinc frame electrode, a novel bifunctional device of ESW‐PZ was realized. It demonstrated good optical/electrical bistability, high electrochemical reversibility, excellent electrochromism, and desirable energy storage performance. A significant energy‐saving efficiency of 16% has been discovered in major cities in China, based on simulation calculations. It also offered a high electric energy recovery. An electric energy of 855 mWh m^−2^ could be reused during each bleaching process, which was equal to 90% of the wasted electric energy. This amount of electric energy can be reused to power small household appliances, thus contributing to the development of near‐zero energy buildings.

## 2 Results and Discussion

### 2.1 Schematic of ESW‐PZ Model and Advantages of Molecular Design

**Figure 1:**
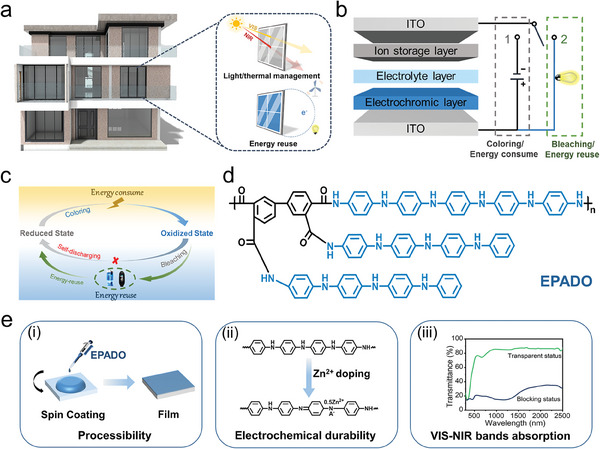
The bifunctional of ESW‐PZ and the advantages of electroactive polyamide of EPADO. a) Schematic illustration of the energy‐saving building equipped with ESW‐PZ and the functions of ESW‐PZ. b) The circuit‐switching strategy of ESW‐PZ for electrochromism and energy reuse. c) The mechanism of electrochromism and energy reuse of ESW‐PZ. d) Molecular structure of EPADO. e) Advantages of EPADO as an electrochromic material including good processibility, electrochemical durability, and VIS‐NIR dual‐band absorption.

A schematic diagram of an energy‐saving building equipped with ESW‐PZ is shown in **Figure** **1**a. During the daytime, when the sun is shining brightly, the solar radiation can be blocked by applying a small voltage to ESW‐PZ, thereby keeping the interior cool and achieving simultaneous modulation of light and temperature conditions. In cloudy and cold weather conditions, the ESW‐PZ can be set to a transparent state, allowing solar radiation to enter the interior and thus creating a comfortable environment. It is noted that ESW‐PZ consumes electric energy unidirectionally during a round‐trip coloring‐bleaching process, and the electric energy involved in the bleaching process was wasted through self‐discharging. Technically, this wasted electric energy could be transferred and stored in other energy storage equipment or used directly to charge small appliances. Therefore, an ingenious circuit‐switching strategy that enabled the separating of coloring and bleaching processes was proposed to achieve both electrochromism and energy reuse. As shown in Figure 1b, when a single‐blade double‐throw switch was connected to the 1 contact, the coloring process was driven by an external power source, and the electric energy was then transferred and stored in ESW‐PZ. As the bleaching state was required, the single‐blade double‐throw switch was connected to the two contacts. The energy stored in ESW‐PZ can be reused again to power small household appliances directly and/or transferred into other energy storage equipment. Its detailed mechanism in the ESW‐PZ is illustrated in Figure 1c.

As the core component of ESW, electrochromic materials need to have reversible electroactivity, outstanding cyclic durability, and wide‐band absorption to realize the bifunction of electrochromism and energy storage. In this work, an electroactive polyamide with dense oligoaniline groups (EPADO) through a three‐step reaction was designed and synthesized (Figure S1, Supporting Information). Its molecular structure is provided in Figure 1d. As a polyaniline derivative, EPADO was expected to have both inherent excellent electro‐optical features and energy storage properties of polyaniline, as well as improved processibility due to its nonconjugated and pendant architecture. The molecular structures of all the intermediate products and EPADO were verified using infrared spectroscopy and NMR spectroscopy (Figures S2–S7, Supporting Information).

XRD tests were performed on EPADO and polyaniline. As shown in Figures S8 and S9 (Supporting Information), EPADO had only one broad peak, while polyaniline had many sharp diffraction peaks, which indicated that polyaniline had higher orientation ordering and crystallinity than EPADO. Therefore, the molecular structure of EPADO bearing two pendants was looser, which was more conducive to the embedding or detachment of the ions. Finally, the energy storage performance of EPADO was higher than previously reported polyaniline‐based electrochromic supercapacitors. As expected, the nonconjugated molecular structure and pendant architecture enabled EPADO to have good solubility in strong organic solvents (e.g., N‐methyl‐2‐pyrrolidone, dimethylsulfoxide, dimethylformamide, and dimethylacetamide). It can be ascribed to the reduced hydrogen‐bond interaction and low rigidity of polymeric main chains. The resultant good solution processibility is beneficial for large‐scale production (Figure 1e(i)). A secondary doping technique of polyaniline‐type materials was applied in this work. EPADO was treated with 1.0 m Zn(CF_3_SO_3_)_2_/ACN solution instead of sulfuric acid to avoid the corrosion to the current collector caused by an acidic solution, thereby improving the electrochemical durability of ESW‐PZ (Figure 1e(ii)). Moreover, the EPADO doped with Zn^2+^ offered a wider voltage window compared to that of H^+^, leading to a higher energy density. In addition, the colored state of EPADO almost achieved a full VIS‐NIR dual‐band absorption (Figure 1e(iii)), providing a theoretical basis for modulating the transmittance of solar radiation. Furthermore, the thermal gravimetric analysis results revealed a good thermostability of EPADO (Figure S10, Supporting Information), indicating its suitability for practical applications in ESW.

### 2.2 Electrochromic and Energy Storage Properties of EPADO/ITO Electrode

**Figure 2:**
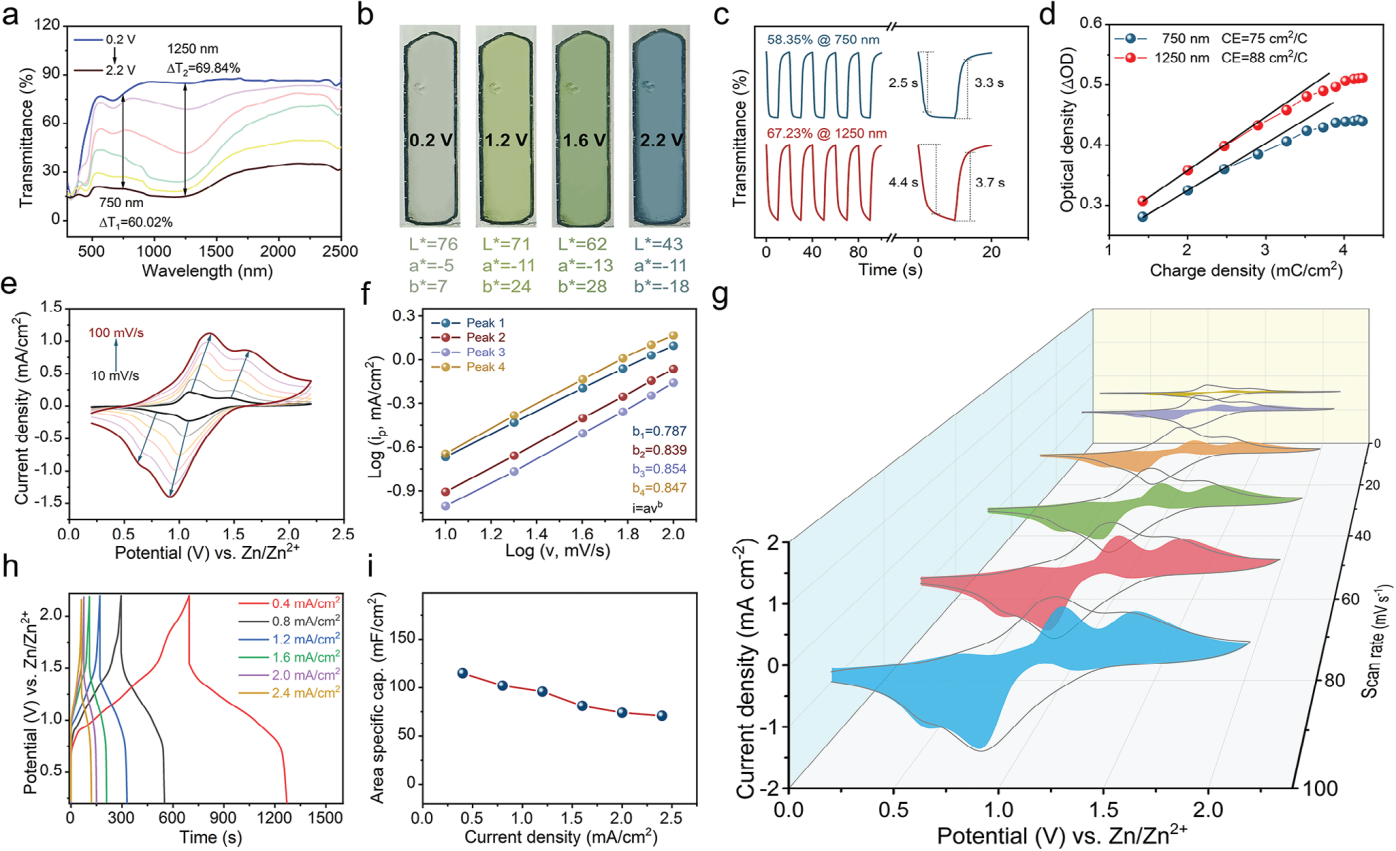
Electrochromic and electrochemical properties of EPADO/ITO electrode. a) Continuous transmittance changes of the EPADO/ITO electrode at different potentials. b) Color switching of the EPADO/ITO electrode at different potentials. c) Dynamic response switching, response time, and d) coloration efficiency plots of the EPADO/ITO electrode at 750 and 1250 nm. e) CV curves of the EPADO/ITO electrode at different scan rates. f) The plot of logi versus logv of the redox peaks in the CV curve. g) The 3D schematic illustrates the contribution of diffusion and capacitive control processes at different scan rates. h) GCD curves of EPADO/ITO electrode. i) Specific capacitances of the EPADO/ITO electrode at different current densities.

Transmittance modulation and energy storage are contradictory for electrochromic energy storage devices. The focus of this work is to compose a new type of electrochromic smart window to improve the indoor environment by transmittance changing. Therefore, the electrochromic performance of the device was prioritized. The loading amount of EPADO depends on the transmittance contrast of the working electrode, which usually should be maintained at more than 50%. In this work, the EPADO/ITO electrodes are fabricated by spin‐coating EPADO DMF solution, and then drying under a warm environment. The content of the active substance on the working electrodes is about 0.8–0.9 mg cm^−2^ calculated by weighing method. The transmittance changes of the EPADO/ITO electrode were monitored by a UV–vis‐NIR spectrophotometer combined with an electrochemical workstation. It exhibited a significant transmittance response in the range of 300–2500 nm (**Figure** **2**a). As the potential increased, the transmittance gradually decreased, with a maximum transmittance of 60.02% at 750 nm and 69.84% at 1250 nm. It displayed distinct color changes at different potentials (Figure 2b): nearly transparent at 0.2 V, light green at 1.2 V, deep green at 1.6 V, and deep blue at 2.2 V. These results evidenced the excellent electrochromic performance of EPADO/ITO electrode. The transmittance switching characteristics were obtained using the chronoamperometry technique with a pulse time of 10 s (Figure 2c). The maximum transmittance was 58.35 and 67.23% at 750 and 1250 nm, respectively. The colored and bleached times required for a 90% transmittance change were 2.5 s/3.3 s and 4.4 s/3.7 s, indicating moderate response speeds.^[^ ^42^, ^43^, ^44^, ^45^ ^]^ The Coloration efficiency (CE) for evaluating electrochromic performance was obtained from the equation: *CE = log(T_b_/T_c_)/ΔQ*, where *T_b_* and *T_c_* are the transmittance values at the beginning and end of the transmittance change, and *ΔQ* is the charge consumption per unit area. As shown in Figure 2d, the CE values of the EPADO/ITO electrode were 75 and 88 cm^2^ C^−1^ at 750 and 1250 nm, respectively. It demonstrated a similar charge utilization efficiency compared to other polymeric electrochromic materials.^[^ ^42^, ^43^, ^44^, ^45^ ^]^ All these results confirmed a satisfactory electrochromic performance of EPADO/ITO electrodes.

The EPADO/ITO electrodes exhibited two distinct pairs of oxidation‐reduction peaks located at 1.10 V/0.79 V and 1.48 V/1.08 V (Figure 2e), evidencing the changes in the redox states during electrochemical processes. The large current responses observed in CV curves indicated an excellent energy storage performance. The dynamic characteristic of charge transfer was calculated from the relationship between current and scan rate in the CV curves based on the equation: *i = av^b^*. The EPADO/ITO electrode exhibited excellent capacitance characteristics with b values of 0.787, 0.839, 0.854, and 0.847 for Peaks 1 to 4 (Figure 2f), respectively. The contributions of the diffusion‐ and capacitance‐controlled processes at different scan rates are shown in Figure 2g. The capacitance contribution gradually increased with the increase in scan rates, from 52.1% at 10 mV s^−1^ to 80.2% at 100 mV s^−1^ (Figure S11, Supporting Information).

Electrochemical impedance spectroscopy (EIS) was used to measure the resistance of EPADO/ITO electrodes (Figure S12, Supporting Information). It was about 81 Ω, comparable to the reported electrochromic electrodes,^[^ ^25^, ^31^, ^35^ ^]^ indicative of its good conductivity. Figure 2h shows the galvanostatic charge/discharge (GCD) curves of EPADO/ITO electrodes. At a current density of 0.4 mA cm^−2^, the charge‐discharge time was ≈1270 s, and it was 120 s at 2.4 mA cm^−2^. The relationship between the areal capacitance and current density is presented in Figure 2i. The maximum specific capacitance was about 115.0 mF cm^−2^, superior to the reported electrochromic energy storage devices in a range of 34.1–65.8 mF cm^−2[^ ^6^, ^24^, ^38^ ^]^ The high capacitive performance of EPADO/ITO indicated its promising prospects for practical application in energy storage devices.

### 2.3 Light/Thermal Management of ESW‐PZ

**Figure 3:**
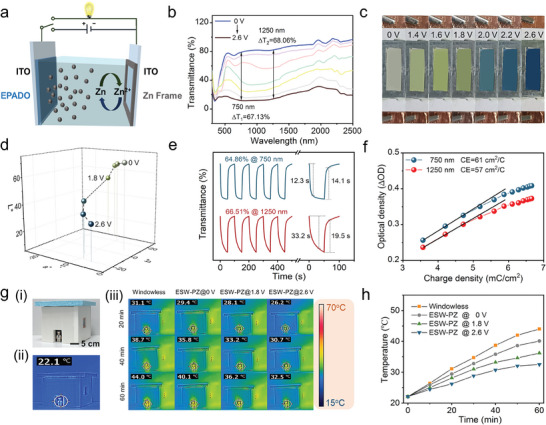
Dual‐band electrochromic performance of ESW‐PZ. a) Schematic of the assembly of ESW‐PZ with the circuit‐switching feature. b) Continuous transmittance change of ESW‐PZ at different voltages. c) Schematic diagram of ESW‐PZ colors at different voltages and d) 3D curves of its L*a*b* values. e) Dynamic transmittance switching and response time, and f) coloration efficiency plots of ESW‐PZ at 750 nm and 1250 nm. g) Digital photograph (i) and infrared thermographic photo (ii) of the house model, (iii) Infrared thermographic photographs of the house model equipped with ESW‐PZ at different voltages under different times of simulated solar irradiation. h) Indoor temperature changes of the house model during 60 min of sunlight exposure.

An ESW‐PZ consisted of an EPADO/ITO working electrode, Zn(CF_3_SO_3_)_2_/ACN electrolyte, a zinc frame counter electrode, and another ITO substrate (**Figure** **3**a). The circuit‐switching design enabled the ESW‐PZ to realize the bifunctions of electrochromism and energy reuse. Under an applied high voltage, EPADO was oxidized immediately with an obvious color change from light to deep blue, while Zn^2+^ ions in the electrolyte were reduced to zinc metal and deposited onto the zinc frame counter electrode. This coloring course was also the charging process of ESW‐PZ. When EPADO was reduced to a transparent state at 0 V, the deposited zinc metal was oxidized to Zn^2+^ ions and transferred into the electrolyte solution. This bleaching process of ESW‐PZ was indeed a discharging process in an energy storage device. The presence of both color‐switching and charging/discharging behaviors indicated that the ESW‐PZ device may be used as both a smart window and a zinc‐ion energy storage device.

To evaluate the electrochromic performance of ESW‐PZ, an electrochemical workstation was used in conjunction with a UV‐VIS‐NIR spectrophotometer. As the voltage increased from 0 to 2.6 V, there was a continuous decrease in the transmittance of the ESW‐PZ in the range of 300–2500 nm (Figure 3b). The maximum transmittance changes at 750 and 1250 nm were 67.13 and 68.06% respectively. This significant contrast in transmittance demonstrated the practicality of ESW‐PZ as a smart window. The ESW‐PZ exhibited a remarkable color change from transparent to green, eventually to dark blue as the applied voltage increased from 0 to 2.6 V (Figure 3c). The L*a*b* values were plotted in a 3D graph (Figure 3d) and the detailed information was provided in Table S1 (Supporting Information).

The transmittance switching characteristics curves of ESW‐PZ were collected using chronoamperometry technology with a pulse time of 50 s (Figure 3e). The maximum transmittance change was determined as about 64.86% at 750 nm and 66.51% at 1250 nm. The colored and bleached times at 750 and 1250 nm were 12.3 s/14.1 s and 33.2 s/19.5 s, respectively. The slow response speed may be attributed to the excellent energy storage capacity of ESW‐PZ and the long pulse time. The CE values of ESW‐PZ were 61 and 57 cm^2^ C^−1^ at 750 and 1250 nm (Figure 3f), respectively.

Considering the desirable light‐blocking ability of ESW‐PZ in both VIS and NIR bands, its thermal insulation performance was thoroughly investigated. First, a house model was equipped with an ESW‐PZ window. The digital photograph and infrared thermographic photo of this house model were presented in Figure 3g(i,ii). The indoor temperature was determined using infrared thermal imagery, which was detected on the face of a cartoon doll. It was carried out on November 08, 2023, in Changchun (43°49N, 125°16E). The room temperature was 22.1 °C. Figure 3g(iii) shows the continuous monitoring of the indoor temperature during the simulated solar irradiation process. More information during different irradiation periods was provided in Figure S13 (Supporting Information). The temperature gradually decreased with the increased voltage regardless of the irradiation time, evidencing the pronounced effect of the applied voltage on the thermal insulation effect of ESW‐PZ. The temperature change curves during simulated solar irradiation were plotted in Figure 3h. The indoor temperature of the house model equipped with ESW‐PZ@2.6 V was decreased by 7.6 °C after 60 min of simulated solar irradiation compared to the ESW‐PZ@0 V, demonstrating an excellent thermal insulation effect of ESW‐PZ through the modulation of solar irradiation transmittance.

### 2.4 Energy‐Saving Simulation of ESW‐PZ

**Figure 4:**
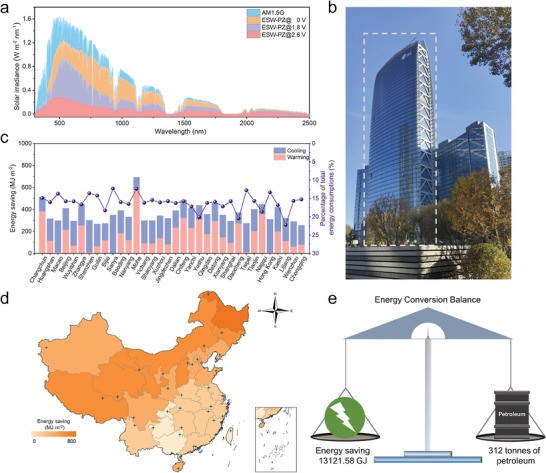
Energy‐saving simulation of ESW‐PZ. a) Solar irradiation transmittance spectra of ESW‐PZ with different voltages. b) Real photographs of Changchun International Finance Centre (IFC). c) Year‐round warming/cooling energy saving and saving percentage of IFC building enveloped with the ESW‐PZ. d) Energy saving mapping of IFC buildings equipped with ESW‐PZ in 34 cities of China. e) Total energy‐saving versus petroleum consumption on the energy conversion balance.

The actual solar irradiance transmittance spectra of ESW‐PZ under different voltages were evaluated with real solar radiation (AM 1.5G). As shown in **Figure** **4**a and Table S2 (Supporting Information), ESW‐PZ exhibited different blocking effects on solar radiation at different voltages. At an applied voltage of 0 V, it was in a transparent state, with *T_VIS_* and *T_NIR_* of 70.6 and 83.5%, respectively, showing the best transmittance performance over the whole wavelength range (74.3%). At 1.8 V, the ESW‐PZ was in a green state with *T_VIS_* and *T_NIR_* of 46.9 and 41.0% respectively, further blocking solar radiation with the whole transmittance of 43.7%. At 2.6 V, EPADO was in its highest oxidation state and ESW‐PZ appeared dark blue, with the lowest transmittance of 15.1% over the entire wavelength range. It blocked most of the visible light (84.8%) and NIR light (85.1%). These results demonstrated that ESW‐PZ possessed a good blocking effect for solar irradiance in both VIS and NIR bands. Therefore, ESW‐PZ fulfilled the requirements for modulating the indoor environment and functioning as a smart window.

To comprehensively demonstrate the energy‐saving capability of building HVAC systems, the optical properties of ESW‐PZ were imported into EnergyPlus to calculate the year‐round energy saving. The Changchun International Finance Center (IFC) with all glass curtain wall structure was chosen as a simulated building (Figure 4b, window‐wall ratio information was in Table S3, Supporting Information), which possessed a 45‐story cuboidal architecture with a length of 60 m, a width of 30 m, and a height of 226 m (Figure S14, Supporting Information). Tables S4 and S5 (Supporting Information) listed the building model information applied in actual building energy simulation and the spectral data of the window samples used in the simulation, respectively. In the energy simulation, the glass curtain wall structure of the IFC building was equipped with ESW‐PZ. Figure 4c shows the energy savings for representative cities in each province (Table S6, Supporting Information provides detailed energy savings data for each city). The ESW‐PZ exhibited better energy‐saving capabilities compared to ordinary commercial low‐emissive glass. Heating energy savings were excellent in colder regions, and cooling energy savings were better in correspondingly hotter regions. For example, in Changchun, located in the northeast of China, there were energy savings of 382.67 MJ m^−2^ per year for heating and 132.32 MJ m^−2^ per year for cooling, contributing to a total energy savings of 514.99 MJ m^−2^ per year (14.90% of total energy consumption). Taipei, located in southern China, there were energy savings of 274.66 MJ m^−2^ per year (12.79% of total energy consumption), with 263.13 MJ m^−2^ per year saved for cooling and 11.53 MJ m^−2^ per year for heating. It verified that the ESW‐PZ had excellent energy‐saving capabilities, fulfilling the expected role of smart windows. Figure 4d shows the energy savings in different provinces of China, compared with commercial low‐emissive glass windows. As shown in Figure 4d, there was almost an upward trend in energy savings from south to north. This was because most buildings in these areas experience significant weather variations including daily and seasonal changes, necessitating dynamic light/thermal management. The ESWs adapted to changing weather conditions could achieve higher energy savings, especially in cold regions. Overall, the simulation results showed that ESW‐PZ saved an average of 366 MJ m^−2^ per year, accounting for ≈16% of the total energy consumption of the IFC building.

According to the average energy saving of 366 MJ m^−2^ per year, the IFC building equipped with ESW‐PZ could save the energy about 13121.6 GJ per year. Such energy would be equivalent to the total energy provided by the complete combustion of 312 tonnes of petroleum (Figure 4e). In other words, this IFC building equipped with ESW‐PZ could reduce CO_2_ emissions by 858 tonnes per year. Compared with commercial low‐emissive glass, the cost of ESW‐PZ in real applications would be higher (Table S7, Supporting Information). However, because the ESW‐PZ is able to regulate the indoor environment by changing itself, it can save a lot of additional energy consumption every year. It has been calculated that it will pay for itself in about four years. From a sustainability point of view, the ESW‐PZ certainly shows great advantages in building construction. Building simulations demonstrated that ESW‐PZ was reliable and practical for saving energy in buildings and enhancing building energy efficiency. The ESW‐PZ may open up a new avenue for achieving the goal of carbon peaking and neutrality.

### 2.5 Energy Storage Properties of ESW‐PZ

**Figure 5:**
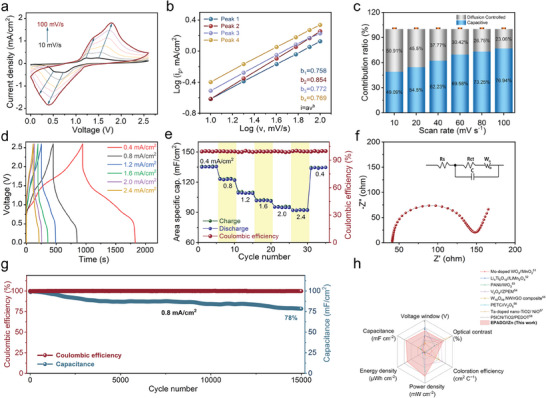
Energy storage properties of ESW‐PZ. a) CV curves of ESW‐PZ at different scanning speeds. b) Plot of log i versus log v of the redox peaks in the CV curve. c) Normalized contribution ratios of capacitive and diffusion control currents at different scan rates. d) GCD curves, e) area‐specific capacitance, and coulombic efficiency for ESW‐PZ. f) Nyquist plot of the ESW‐PZ. g) 15000 long‐cycle test of the ESW‐PZ at a current density of 0.8 mA cm^−2^. h) Radar plot of the performance comparison of the ESW‐PZ with other electrochromic energy storage devices.

The energy storage performance of ESW‐PZ was investigated by conducting CV test (**Figure** **5**a). The scan rates ranged from 10 to 100 mV s^−1^. The ESW‐PZ possessed a wide voltage window of 2.6 V, enabling it to serve as a power source for small household electric appliances. The area of the CV curve increased with the increase in scan rate. The b values of Peak 1, Peak 2, Peak 3, and Peak 4 were calculated to be 0.758, 0.854, 0.772, and 0.769 respectively, which was consistent with a pseudocapacitive behavior (Figure 5b). Figure 5c provides the detailed capacitance contribution rate of the ESW‐PZ. As the scan rate increased, the capacitance contribution of ESW‐PZ became larger and reached 76.9% at 100 mV s^−1^ (Figure S15, Supporting Information showed the 3D schematic of the contribution of diffusion and capacitive control processes at different scan rates.).

Figure 5d shows the GCD curves of ESW‐PZ at the current densities ranging from 0.4 to 2.4 mA cm^−2^. The equilateral triangle GCD curves at all the current densities demonstrated the reversible charge/discharge behavior of ESW‐PZ and its excellent electrochemical dynamics. At a current density of 0.4 mA cm^−2^, the charge/discharge time was as long as 1827 s, indicating the strong energy storage capability of ESW‐PZ. The transmittance changes corresponding to the charging/discharging processes of ESW‐PZ were also provided in Figure S16 (Supporting Information). At 750 and 1250 nm, the transmittance of ESW‐PZ continuously decreased during the charging process, then gradually increased during the discharging process, and finally returned to the original transmittance level. The periodic coordinated fluctuations observed in the GCD curves, along with the simultaneous transmittance response, further highlight the effectiveness of ESW‐PZ. The charge/discharge rate performance of ESW‐PZ was also studied. Figure 5e displayed the area‐specific capacitance and Coulombic efficiency of ESW‐PZ at various current densities, calculated based on the GCD curve. The specific areal capacitance at different scanning rates is shown in Figure S17 (Supporting Information). The maximum areal capacitance at 0.4 mA cm^−2^ was 135.8 mF cm^−2^, which was higher than the capacitance of previously reported polyaniline‐based electrochromic supercapacitors.^[^ ^46^, ^47^, ^48^, ^49^, ^50^ ^]^ Moreover, the ESW‐PZ maintained nearly 100% of coulombic efficiency, indicating its excellent capacitance storage characteristics and electrochemical reversibility.

Figure 5f displays the EIS Nyquist plot and the simulated equivalent circuit. The resistance of ESW‐PZ at 0 V was ≈109 Ω, indicative of good conductivity. Furthermore, Figure S18 (Supporting Information) presented the Ragone plot of ESW‐PZ, revealing an impressive energy density of 127.5 µWh cm^−2^. This result further confirmed the excellent energy storage properties of ESW‐PZ. Figure 5g exhibited the results of the long‐term cycling tests of ESW‐PZ for up to 15000 cycles at 0.8 mA cm^−2^. The capacity retention was ≈100%. Even after 15000 cycles, it still maintained an acceptable capacity retention rate of 78%. SEM images and EIS tests before and after cycling were shown in Figures S19 and S20 (Supporting Information). As a result of long‐term repeating doping/de‐doping, the material collapses internally and the polymer undergoes decomposition, yielding small molecules. Moreover, part of the Zinc ions were immobilized to the surface of the film through the complexation from EPADO. This resulted in the production of some fine particles on the electrode surface after cycling and also the impedance of the device was significantly higher compared to the previous one. The electrochemical performance of ESW‐PZ was compared with other reported electrochromic energy storage devices. As shown in Figure 5h, ESW‐PZ possessed a voltage window of 2.6 V, a high transmittance contrast of 67.1%, a moderate CE of 61 cm^2^ C^−1^, a specific capacitance of up to 135.8 mF cm^−2^, a good power density of 3.1 mW cm^−2^ and a high energy density of 127.5 µWh cm^−2^. They were close to or higher than most reported electrochromic energy storage devices, including Mo‐doped WO_3_//MnO_2_,^[^ ^51^ ^]^ Li_4_Ti_5_O_12_//LiMn_2_O_4_,^[^ ^52^ ^]^ PANI//WO_3_,^[^ ^53^ ^]^ V_2_O_5_//ZPEM,^[^ ^54^ ^]^ W_18_O_49_NW/rGO composite,^[^ ^55^ ^]^ PETC//V_2_O_5_,^[^ ^56^ ^]^ Ta‐doped nano‐TiO_2_/ NiO^[^ ^57^ ^]^ and P5ICN/TiO_2_/PEDOT.^[^ ^58^ ^]^ Table S7 (Supporting Information) lists all detailed data and comparisons with these reported devices.

### 2.6 Energy‐Reuse Performance of ESW‐PZ

**Figure 6:**
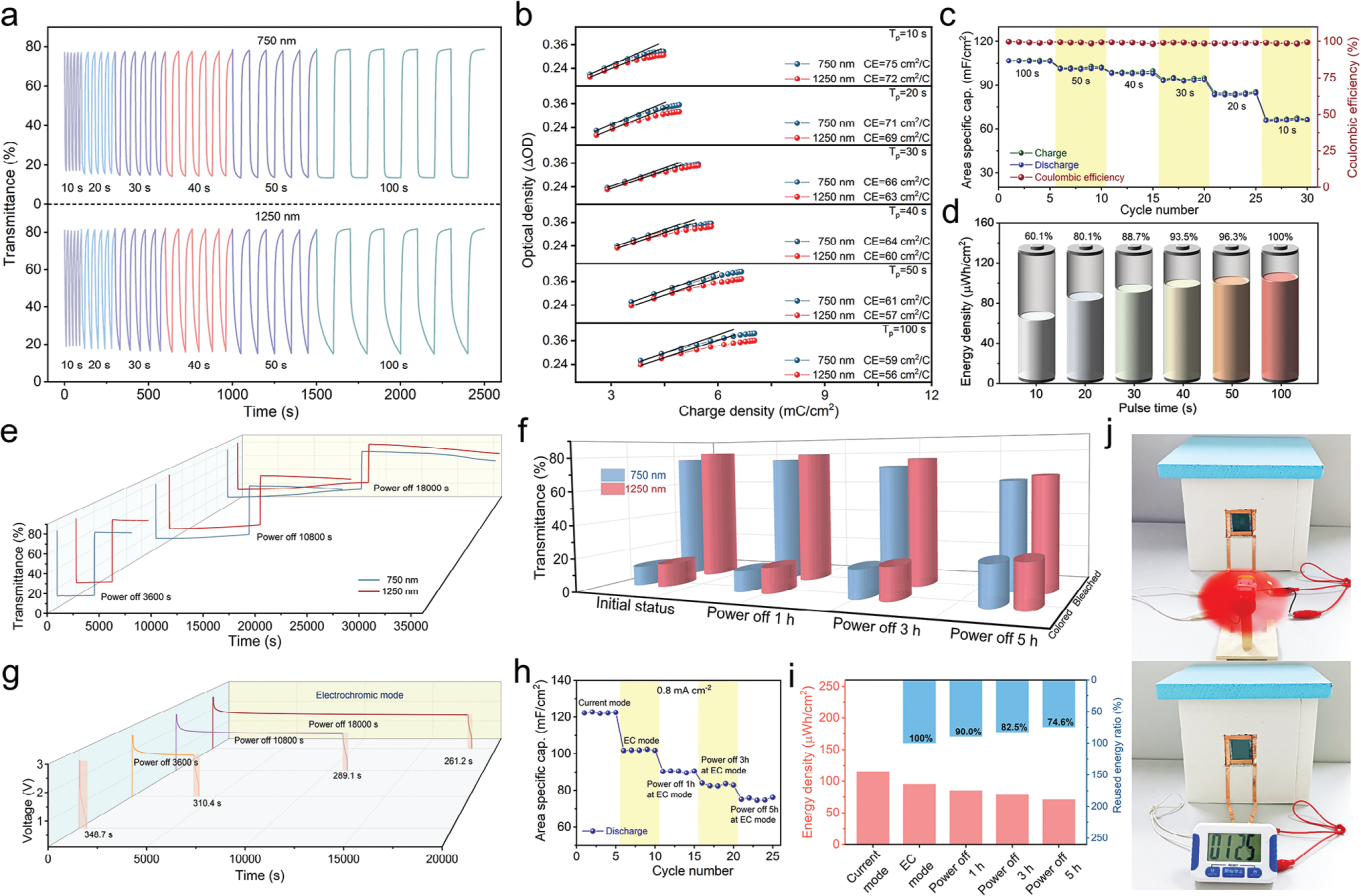
Energy‐reuse performance of ESW‐PZ. a) Dynamic transmittance switching at different pulse times and b) CE plots of ESW‐PZ at 750 and 1250 nm. c) Rate performance and d) energy density of ESW‐PZ at different pulse times. e) Optical memory capability of ESW‐PZ at 750 and 1250 nm with different resting times and f) 3D curves of transmittance after corresponding resting times. g) Comparison of the discharge times of ESW‐PZ in electrochromic mode after different resting times and the calculated h) area‐specific capacitance and i) energy density. j) Power supply for the small fan and stopwatch using the remaining electric energy in the ESW‐PZ after 5 h of resting.

The functioning of ESWs requires electric energy to adjust their light transmittance. Once the light‐blocking task is complete, they become energy storage devices, and the stored energy theoretically can be reused to power household appliances. However, it remained unclear how much electric energy could be retained in ESWs after light‐blocking management. Hence, comprehensive measurements of chronoamperometry and rate performance were conducted on ESW‐PZ for assessment of energy‐reuse properties. The resultant data in **Figure** **6** illustrated the relationship between the electrochromism and energy storage of ESW‐PZ at 2.6 V over different periods. Figure 6a shows the transmittance changes of ESW‐PZ under different pulse durations of 10, 20, 30, 40, 50, and 100 s at 2.6 V. The upper and lower side panels of Figure 6a plotted the continuous transmittance changes of ESW‐PZ at wavelengths of 750 and 1250 nm, respectively, under alternating applied voltages of 0 and 2.6 V. The transmittance contrast of ESW‐PZ gradually increased as the pulse duration lengthened. At 750 and 1250 nm, the transmittance contrast of ESW‐PZ powered for 50 s was 64.8% and 66.5%, respectively, which was pretty close to that of ESW‐PZ powered for 100 s (65.1 and 67.0%). Therefore, ESW‐PZ powered for 50 s met the requirement of the daily indoor environment modulation.

Figure 6b displays the CE values of ESW‐PZ with varying pulse times. They gradually decreased and finally stabilized as the pulse time increased. In the mean time, an increase in charge density was observed, indicating a gradually increased electrical energy stored in the ESW‐PZ, which was consistent with the chronoamperometric results. Compared to ESW‐PZ with a pulse time of 100 s, there was no significant difference in the charge density of ESW‐PZ with a pulse time of 50 s. This demonstrates that 50 s is a reasonable pulse time for ESW‐PZ. Moreover, the area‐specific capacitances of ESW‐PZ under constant voltage modulation mode (electrochromic mode) were collected in Figure 6c. Regardless of the pulse duration of constant voltage charging (10 to 100 s), ESW‐PZ maintained a coulombic efficiency of ≈100%, showing its excellent electrochemical dynamics. It was worth noting that the area‐specific capacitance of ESW‐PZ powered for 50 and 100 s was 101.6 and 106.9 mF cm^−2^, respectively, with little difference. The energy densities of ESW‐PZ with different pulse times at 2.6 V were calculated from Figure 6c and presented in Figure 6d, which continuously increased as the pulse time increased. The energy density of ESW‐PZ powered for 50 s was about 95.3 µWh cm^−2^, which was ≈95% of that for 100 s. These results indicated that the ESW‐PZ upon 2.6 V for 50 s retained almost all of its energy stored. This explains why the ESW‐PZ only required a 50 s‐charge when used as a smart window.

Due to the memory capacity of electrochromic materials’ spectra, the running ESWs worked without being maintained by voltage all the time. Their spectral state underwent a slight decay phenomenon, attributed to the self‐discharging/self‐charging effect of the electrochromic substance upon the external light and heat. Therefore, the relationship between transmittance attenuation and electric energy was investigated in detail for the ESW‐PZ. As shown in Figure 6e, the ESW‐PZ was initially powered at 2.6 V for 50 s to achieve full coloration, followed by continuous spectral testing without any voltage for 1, 3, and 5 h. Subsequently, ESW‐PZ was powered at 0 V for 50 s, resulting in complete bleached. Subsequently, similar spectral testing was conducted without any voltage for 1, 3, and 5 h. The transmittance values of ESW‐PZ after different resting times are shown in Figure 6f. The longer the resting time, the more the transmittance attenuation was found. After 1, 3, and 5 h of coloring state, the transmittance attenuation at 750/1250 nm was 0.7%/1.1%, 4.8%/5.2%, and 11.0%/10.4%, respectively. After 1, 3, and 5 h of bleaching state, the transmittance attenuation was 0.8%/1.4%, 4.9%/4.7%, and 13.0%/14.1%, respectively. Furthermore, Figure S21 (Supporting Information) provided the transmittance spectra of ESW‐PZ in the colored state after different resting times over the range of 300–2500 nm. The spectral memory capacity of ESW‐PZ essentially met the requirements for daily usage of light/thermal management. These results demonstrated that ESW‐PZ could achieve simultaneous modulation of VIS light and NIR radiation over a long period, further proving its excellent optical bistability and enormous potential for energy‐saving applications in the field of green buildings.

A combination of electrochromic mode with constant current discharging mode was utilized to determine the residual electric energy of ESW‐PZ after optical modulation with different resting times. Specifically, ESW‐PZ was modulated in constant voltage mode (electrochromic mode) at 2.6 V, then powered off for 1, 3, and 5 h, and subsequently discharged in constant current mode at 0.8 mA cm^−2^ (energy discharge mode) to calculate the stored energy and specific capacitance. As shown in Figure 6g, after a longer resting time, the discharge time of ESW‐PZ became shorter, indicating a lower stored capacitance due to self‐discharge. All the area‐specific capacitances and energy densities of ESW‐PZ under different statuses were supplied in Figure 6h,i. ESW‐PZ could possess 90.0%, 82.5%, and 74.6% of the electric energy after resting for 1, 3, and 5 h, respectively, which represented truly reusable electrical energy to power small electronic products. Figure 6j demonstrates the retained electric energy in the ESW‐PZ after a 5‐h rest could power a small fan and stopwatch, indicating its unique energy‐reuse feature and practicality. The transmittance modulation and energy‐reuse process was clearly recorded in Videos S1 and S2 (Supporting Information).

Furthermore, we calculated the reusable electric energy of ESW‐PZ installed in the IFC building to modulate the indoor environment. During the calculation process, ESW‐PZ was assessed after 3 h of resting based on its daily usage of three times for light/thermal management and then charging other small appliances. The reusable electric energy was about 111.09 GJ m^−2^ per year. This amount of energy was equivalent to the full combustion of 2.64 tonnes of petroleum which yields a CO_2_ emission of 7.26 tonnes. The energy‐reused feature of ESW‐PZ further advances energy conservation and emission reduction, and enhances the building's energy efficiency.

## 3 Conclusion

An energy‐reuse featured ESW has been developed by coupling a novel electroactive polyamide with a zinc framework electrode. An ingenious circuit‐switching strategy is applied to separate the coloring and bleaching processes, enabling the realization of electrochromism and energy reuse of ESW‐PZ. It exhibits desirable indoor light/thermal management due to its VIS‐NIR dual‐band electrochromic performance of polyamide. Based on building energy simulations, ESW‐PZ as building envelopes can save an average annual HVAC energy consumption of 366 MJ m^−2^, which is about 16% of the total building energy consumption. Moreover, the outstanding energy storage characteristics of ESW‐PZ endow it with a high capability for energy reuse. During each bleaching process, 74.6–90.0% of electric energy stored in ESW‐PZ can be reused to power small household appliances directly and/or transferred into other energy storage equipment. This technology is expected to inspire the further exploitation of energy‐saving ESW exploitation for advancing the goal of near‐zero energy consumption building.

## Conflict of Interest

The authors declare no conflict of interest.

## Supporting information



Supporting Information

Supplemental Video 1

Supplemental Video 2

## Data Availability

The data that support the findings of this study are available from the corresponding author upon reasonable request.
