# Supplementary material for: Reusing the Wasted Energy of Electrochromic Smart Window for Near‐Zero Energy Building
Source: Adv Sci (Weinh). 2024 Sep 16;11(42):2406232. doi: 10.1002/advs.202406232 (PMC11558134; doi:10.1002/advs.202406232)
Supplement: Supplementary file 1 — Supporting Information [file ADVS-11-2406232-s001.docx]

**Supplementary Information**

**Reusing the Wasted Energy of Electrochromic Smart Window for Near-Zero Energy Building**

Yunfei Xie^a^, Ruonan Huang^a^, Meini Li^a^, Ningzhi Cao^a^, Xiaoteng Jia^b,*^, Caiyun Wang^c,*^, Danming Chao^a,*^

^a^College of Chemistry, Jilin University, Changchun, 130012, China

^b^State Key Laboratory of Integrated Optoelectronics, College of Electronic Science and Engineering, Jilin University, Changchun 130012, China

^c^Intelligent Polymer Research Institute, Faculty of Engineering and Information Sciences, Innovation Campus, University of Wollongong, North Wollongong, NSW 2500, Australia.

E-mail: xtjia@jlu.edu.cn (X Jia); [caiyun@uow.edu.au](mailto:caiyun@uow.edu.au) (C Wang); chaodanming@jlu.edu.cn (D Chao)

**Experimental section**

**Materials**

Zinc trifluoromethanesulfonate (Zn(CF_3_SO_3_)_2_), [5,5'-Biisobenzofuran]-1,1',3,3'-tetraone were purchased from Shanghai Macklin Biochemical Co., Ltd. 4-aminodiphenylamine, *N*,*N’*-dimethylformamide (DMF), *p*-phenylenediamine, ammonium persulfate (APS), 4-Dimethylaminopyridine (DMAP), 1-(3-Dimethylaminopropyl)-3-ethylcarbodiimide hydrochloride (EDC), and acetone were purchased from Energy Chemical. Ethanol (EtOH), acetonitrile (ACN), and dichloromethane (DCM) were obtained from Sinopharm Chemical Reagent Co., Ltd. Other reagents and solvents were used as received from commercial sources without further treatment. The indium tin oxide glass (ITO, 10 Ω cm^-2^) was obtained from Zhuhai Kaivo Optoelectronic Technology Co., Ltd.

**Apparatus**

^1^H NMR and ^13^C NMR spectra of the synthesized molecules were received on a Bruker Avance NEO 400 system (400 MHz). The chemical shifts were presented in ppm using tetramethylsilane (TMS) as the internal standard. BRUKER VECTOR 22 Spectrometer was used to gather the Fourier-transform infrared spectra (FTIR) in the range of 4000-400 cm^-1^. The molecular weight information of the polymer was determined on a Shimadzu gel permeation chromatography unit equipped with a Shimadzu GPC-802D gel column and SPDM10AVP detector. The thermal property was evaluated by thermogravimetric analysis (TGA) on PerkinElmer PYRIS 1 TGA in the temperature range of 100-600^o^C with a rate of 10^o^C min^-1^ under N_2_ atmosphere. The transmittance spectra were monitored using UV-Vis-NIR spectra on a UH4150 spectrometer. The data of CIE *L*a*b** were obtained from a high-quality portable colorimeter (Shenzhen 3nh Co., Ltd., Shenzhen, China). Infrared photos were taken with an infrared thermal imager (FLIR Systems Inc). The cyclic voltammetry (CV) and electrochemical impendence spectroscopy (EIS) were collected on a CHI 660E Electrochemical Workstation (CH Instruments, USA) using a three-electrode electrochemical cell, which was constituted with a Zn/Zn^2+^ reference electrode, a platinum counter electrode, and a polymer/ITO working electrode. The software of Zview optimized the equivalent circuit in EIS measurement.

**Synthetic procedure**

**Synthesis of 3',4-bis((4-(phenylamino)phenyl)carbamoyl)-[1,1'-biphenyl]-3,4'-dicarboxylic acid (DAA)**

3.13 g (16.99 mmol) of 4-aminodiphenylamine was dissolved in 25 mL of DMF and sonicated. 2.00 g (6.80 mmol) of [5,5'-Biisobenzofuran]-1,1',3,3'-tetraone was added to 40 mL of DMF and sonicated to make it well dispersed. During stirring, the suspension of [5,5'-Biisobenzofuran]-1,1',3,3'-tetraone was slowly dropped into a solution of 4-aminodiphenylamine. It was fully reacted under a nitrogen atmosphere for 3 h and poured into an appropriate amount of water. Filtered, and washed with distilled water and DCM respectively until colorless and dried in a vacuum oven, the solid product DAA was obtained (83% yield). FTIR (KBr, cm^-1^): 3383 (ν_N-H_), 3200-2500 (ν_O-H_), 3031 (ν_Ar-H_), 1707, 1652 (ν_C=O_), 1597, 1514 (-C=C- stretch in the benzene), 1311 (ν_C-N_). ^1^H NMR (400 MHz, DMSO-*d*_6_): δ 13.05 (O-H), 10.50, 10.45 (CO-NH), 8.50-7.50 (Ar-H), 7.50-6.50 (N-H). ^13^C NMR (101 MHz, CDCl_3_): δ 168.29, 168.10, 166.80, 144.65, 139.46, 133.01, 129.68, 129.47, 123.05, 121.42, 121.36, 119.51, 118.33, 116.30, 115.29, 114.15.

**Synthesis of electroactive polyamide acid (PDA)**

2.00 g (3.02 mmol) of DAA and 0.33 g (3.02 mmol) of p-phenylenediamine were dissolved in 18 mL of DMF. 35 mL of DMF, 5 mL of distilled water, and 5 mL of concentrated hydrochloric acid were mixed and added slowly dropwise to the solution of p-phenylenediamine. 1.38 g (6.04 mmol) of APS was dissolved in 12 mL of 1 mol/L hydrochloric acid solution, sonicated to make it homogeneous, and then added slowly dropwise to the above DMF solution. The reaction was stopped after 15 h and poured into an appropriate amount of water. Filtered and cleaned with distilled water, DCM, and EtOH respectively. Vacuum drying was done to obtain electroactive polyamidoic acid PDA (86% yield). FTIR (KBr, cm^-1^): 3417 (ν_N-H_), 3100-2500 (ν_O-H_), 3045 (ν_Ar-H_), 1720, 1649 (ν_C=O_), 1585, 1509 (-C=C- stretch in the benzene), 1313 (ν_C-N_). ^1^H NMR (400 MHz, DMSO-*d*_6_): δ 13.01 (O-H), 10.70-10.00 (CO-NH), 8.70-7.50 (Ar-H), 7.50-6.20 (N-H).

**Synthesis of electroactive polyamide bearing dense oligoaniline groups (EPADO)**

Add 0.30 g (0.38 mmol) PDA and 0.01 g (0.07 mmol) DMAP to 10 mL DMF. 0.28 g (0.75 mmol) Aniline tetramer and 0.43 g (2.28 mmol) EDC were added to another 10 mL of DMF and slowly added dropwise to the DMF solution of PDA under nitrogen atmosphere. The reaction was carried out for 12 h. The mixture was poured into an appropriate amount of distilled water. Filtered and washed with distilled water, DCM and acetone respectively until colorless. The final product EPADO was obtained after drying (77% yield). FTIR (KBr, cm^-1^): 3396 (ν_N-H_), 3041 (ν_Ar-H_), 1655 (ν_C=O_), 1600, 1511 (-C=C- stretch in the benzene), 1299 (ν_C-N_). ^1^H NMR (400 MHz, DMSO-*d*_6_): δ 10.45-10.05 (CO-NH), 8.50-7.40 (Ar-H), 7.40-6.50 (N-H). GPC data: Mn: 42.2 K, PDI: 2.08.

**The assembly of ESW-PZ**

EPADO (16 mg) was added to 2 mL of DMF solution with sonication of 60 min. The resulting solution was added dropwise onto the ITO substrate and spin-coated at 2500 r/s for 20 s to obtain a homogeneous polymeric film, which was then placed in a 65°C vacuum oven overnight, yielding a EPADO/ITO working electrode.

The ESW-PZ was assembled using a EPADO/ITO working electrode, a zinc frame/ITO counter electrode, and an intermediate 1.0 M Zn(CF_3_SO_3_)_2_/ACN electrolyte. Appropriate encapsulation was used to avoid contamination and leakage of the device.


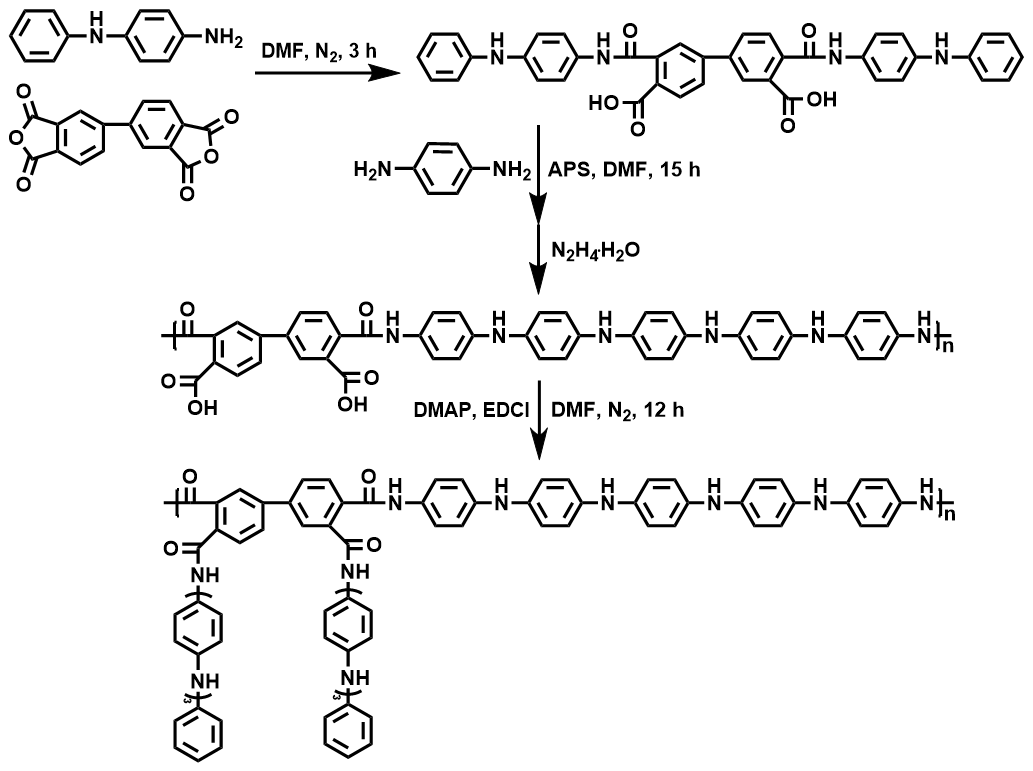


**Figure S1.** Synthetic route of EPADO.





**Figure S2.** FTIR spectra of DAA.





**Figure S3.** ^1^H NMR spectrum of DAA.





**Figure S4.** ^1^C NMR spectrum of DAA.





**Figure S5.** FTIR spectra of PDA.





**Figure S6.** FTIR spectra of EPADO.





**Figure S7.** ^1^H NMR spectrum of PDA and EPADO (PDA at the top and

EPADO at the bottom).





**Figure S8.** The XRD spectrum of EPADO.





**Figure S9.** The XRD spectrum of polyaniline.





**Figure S10.** TGA curve of EPADO.





**Figure S11.** Normalized contribution ratios of capacitive and

diffusion control currents at different scan rates.





**Figure S12.** Nyquist plot of the EPADO/ITO electrode.


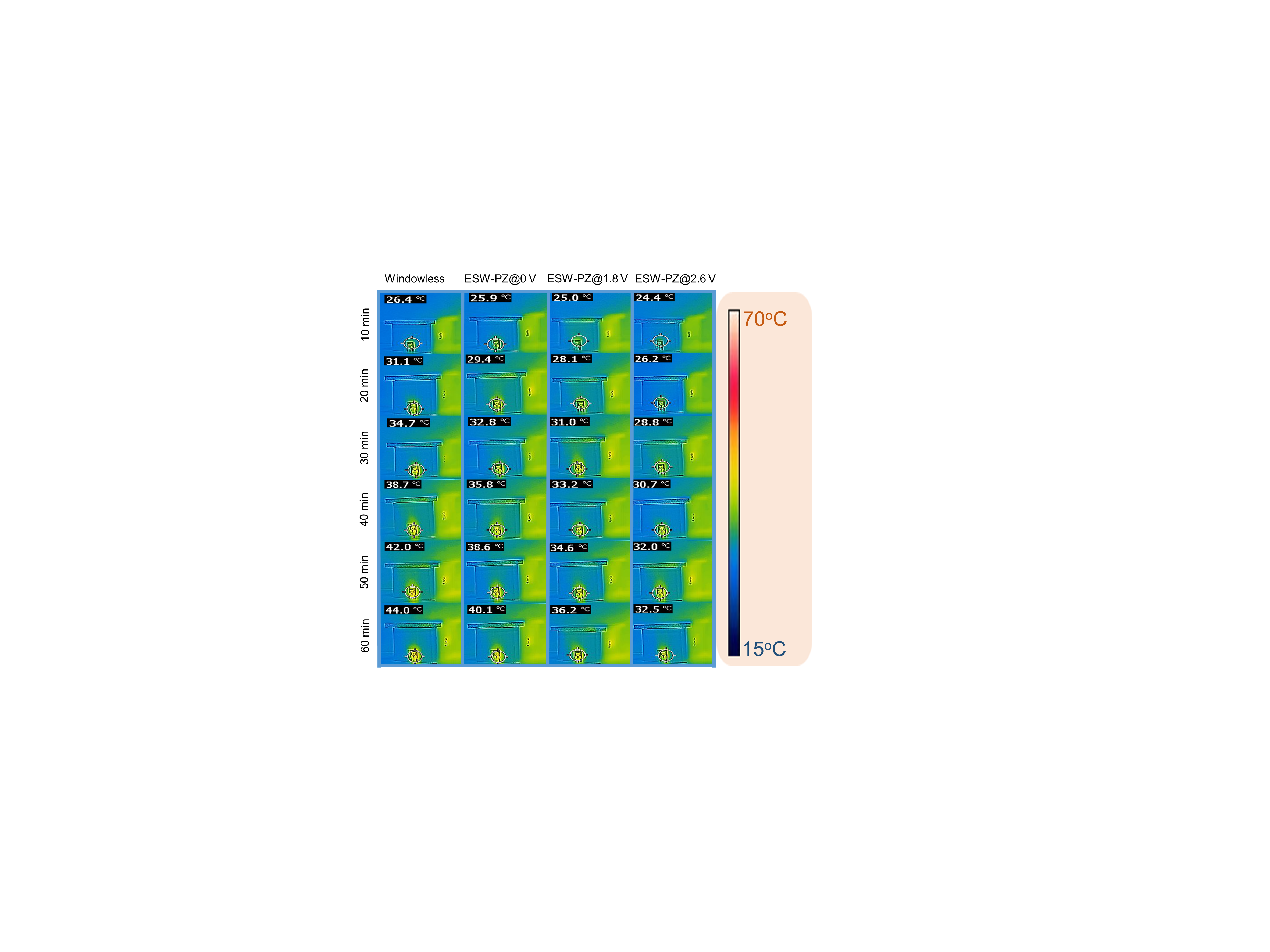


**Figure S13.** Infrared thermal imagery at different times of irradiation.


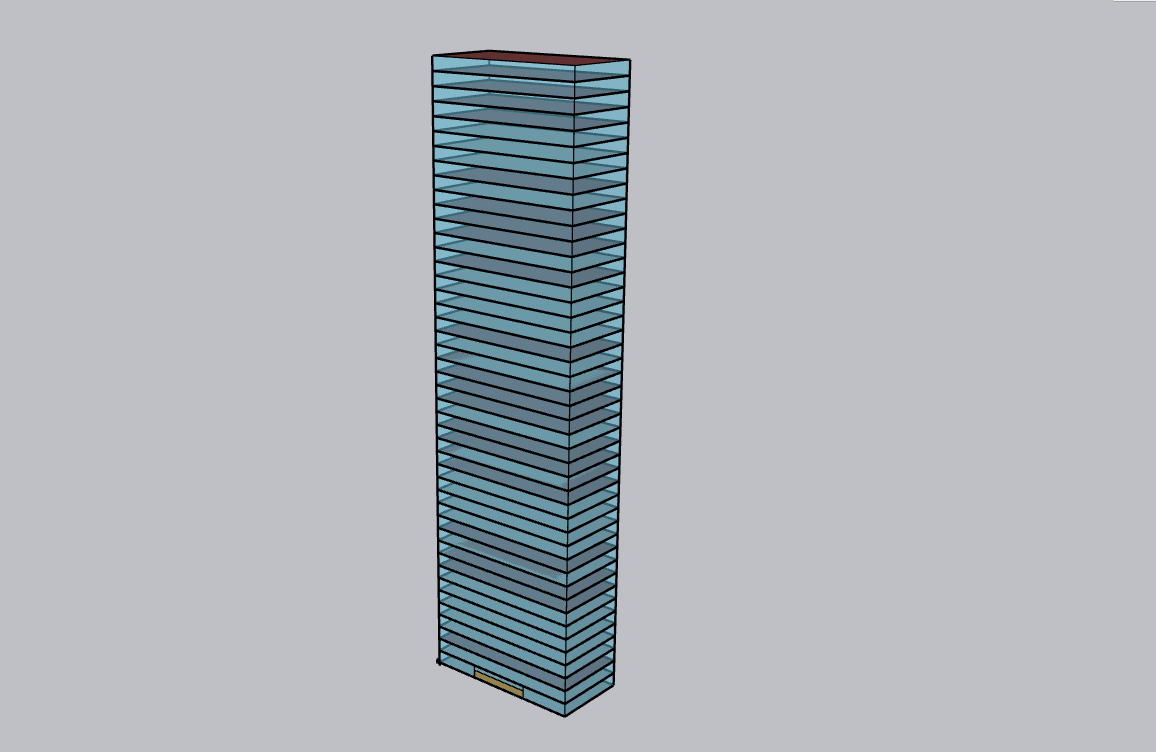


**Figure S14.** A real building model of the IFC.





**Figure S15.** 3D schematic of the contribution of diffusion and capacitive control processes of ESW-PZ at different scan rates.





**Figure S16.** Combination of GCD curves and transmittance curves at 750 nm and 1250 nm for ESW-PZ.





**Figure S17.** Specific capacitance values of the ESW-PZ at different current densities.





**Figure S18.** The Ragone plot of the ESW-PZ.


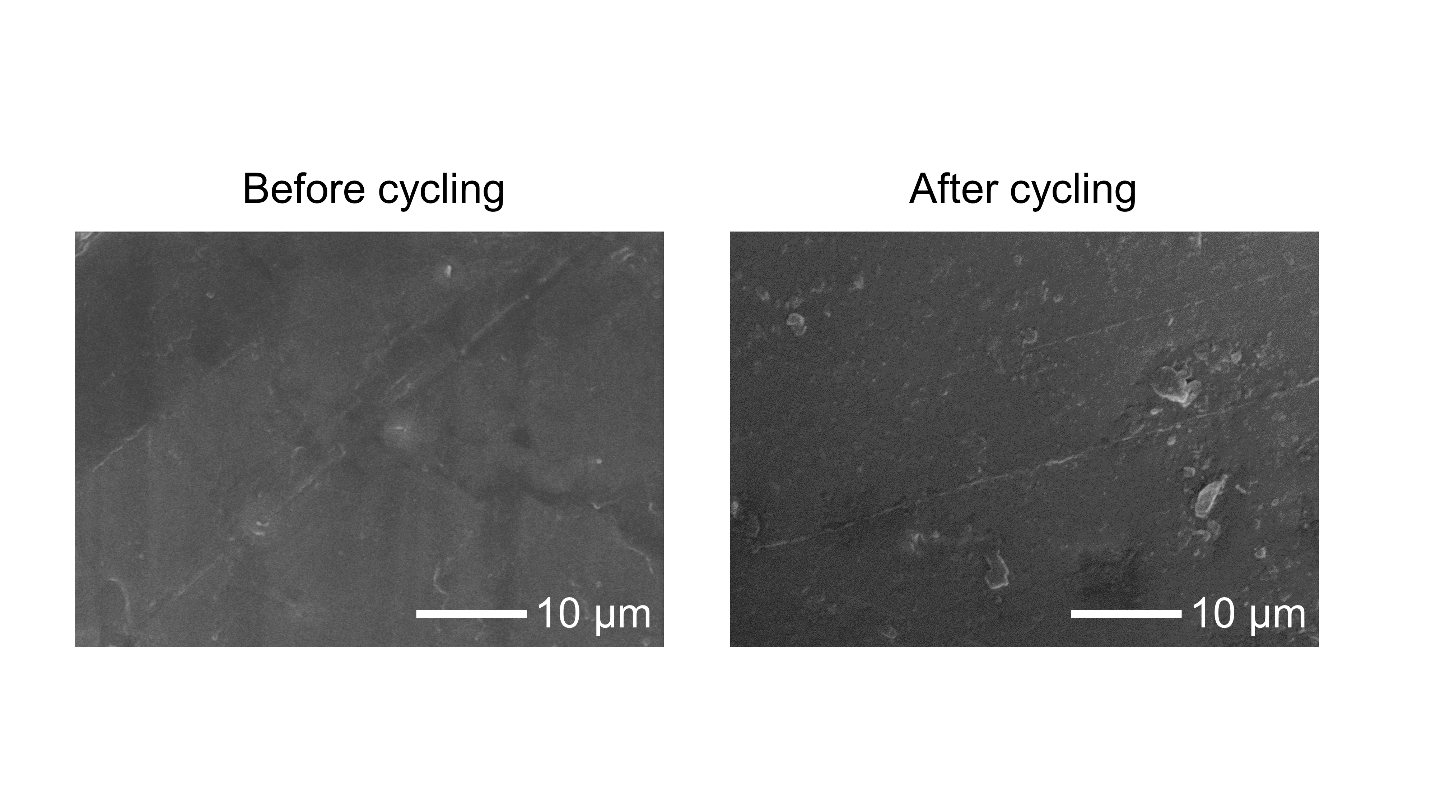


**Figure S19.** SEM images before and after 15000 cycles.





**Figure S20.** Nyquist plot before and after 15000 cycles.





**Figure S21.** Transmittance spectra of ESW-PZ in the colored state after being rested for different times in the range of 300-2500 nm.

**Supplementary Table S1**

The *L*a*b** value of ESW-PZ at different voltages.

| Voltage | *L** | *a** | *b** |
| --- | --- | --- | --- |
| 0 V | 70.08 | -4.21 | 7.37 |
| 1.4 V | 66.54 | -10.75 | 21.51 |
| 1.6 V | 63.34 | -13.65 | 29.60 |
| 1.8 V | 51.25 | -17.78 | 35.34 |
| 2.0 V | 44.12 | -11.21 | -12.72 |
| 2.2 V | 37.20 | -9.32 | -19.90 |
| 2.6 V | 32.06 | -6.54 | -21.16 |

**Supplementary Table S2**

Integrated solar irradiance transmittance (T) of ESW-PZ at different voltages for visible (400-780 nm), near-infrared (780-2500 nm), and full solar spectrum (400-2500 nm).

| Voltage | *T_VIS_* (%) | *T_NIR_* (%) | *T_FULL_* (%) |
| --- | --- | --- | --- |
| 0 V | 70.6 | 83.5 | 74.3 |
| 1.8 V | 46.9 | 41.0 | 43.7 |
| 2.6 V | 15.2 | 14.9 | 15.1 |

The calculation was based on the equation: $T=\frac{\int T\left( \lambda\right)\psi\left( \lambda\right)d\lambda}{\int\psi\left( \lambda\right)d\lambda}$.

Where *T(λ)* is the transmittance at a wavelength of *λ*, and *ψ(λ)* is the solar irradiance at 1.5 air mass.

**Supplementary Table S3**

Simulation of the actual window-to-wall ratio of the building.

|  | Total |
| --- | --- |
| Total building area (m^2^) | 40680.0 |
| Area of one-storey building (m^2^) | 904.0 |
| Gross Wall Area (m^2^) | 40050.0 |
| Window Opening Area (m^2^) | 35851.3 |
| Gross Window-Wall Ratio (%) | 89.5 |

**Supplementary Table S4**

| Items | Specifications |
| --- | --- |
| Exterior walls | Stucco, heavy-weight concrete, wall insulation, gypsum board |
| Roof | Roof membrane, roof insulation, metal decking |
| HVAC | Ideal-loads-air-system served by district cooling and heating sources, thermostat setpoint 24°C cooling/21°C heating |

Information of the building model applied in actual building energy simulation.

|  | Total |
| --- | --- |
| Total building area (m^2^) | 40680.0 |
| Area of one-storey building (m^2^) | 904.0 |
| Gross Wall Area (m^2^) | 40050.0 |
| Window Opening Area (m^2^) | 35851.3 |
| Gross Window-Wall Ratio (%) | 89.5 |

|  | Total |
| --- | --- |
| Total building area (m^2^) | 40680.0 |
| Area of one-storey building (m^2^) | 904.0 |
| Gross Wall Area (m^2^) | 40050.0 |
| Window Opening Area (m^2^) | 35851.3 |
| Gross Window-Wall Ratio (%) | 89.5 |

**Supplementary Table S5**

Spectral data of windows samples used in the simulations.

| Windows glass spetral data set name | ESW-PZ | | Commercial low-E glass |
| --- | --- | --- | --- |
|  | Coloed | Bleached |  |
| Thickness (mm) | 6 | 6 | 6 |
| *T_VIS_* (%) | 15.2 | 70.6 | 87 |
| R*_VIS-Front_* (%) |  |  | 4.3 |
| R*_VIS-Back_* (%) |  |  | 7.2 |
| *T_FULL_* (%) | 15.1 | 74.3 | 66 |
| R*_FULL-Front_* (%) |  |  | 18 |
| R*_FULL-Back_* (%) |  |  | 20 |
| *T_NIR_* (%) | 14.9 | 83.5 |  |
| εNIR |  |  | 0.84 |
| εNIR-Back |  |  | 0.1 |

**Supplementary Table S6**

Annual and percentage energy savings for buildings using ESW-PZ.

|  | Waming energy saving (MJ/m^2^) | Cooling energy saving (MJ/m^2^) | Total energy saving (MJ/m^2^) | Percentage of total  energy consumptions (%) |
| --- | --- | --- | --- | --- |
| Changchun | 382.67191 | 132.3218 | 514.99371 | 14.90019 |
| Huangshan | 114.83685 | 201.98382 | 316.82067 | 16.00973 |
| Macao | 8.8391 | 289.55663 | 298.39573 | 13.73236 |
| Beijing | 213.74674 | 197.67865 | 411.42539 | 15.75235 |
| Wuyishan | 69.99865 | 225.14742 | 295.14607 | 15.77537 |
| Zhangye | 254.77888 | 191.67213 | 446.45101 | 16.61793 |
| Shenzhen | 10.39596 | 291.34045 | 301.7364 | 13.58979 |
| Guilin | 65.2809 | 202.71888 | 267.99978 | 14.21592 |
| Bijie | 118.13663 | 155.58067 | 273.7173 | 18.26103 |
| Sanya | 0.96629 | 349.2382 | 350.20449 | 12.2954 |
| Baoding | 182.43775 | 209.01708 | 391.45483 | 15.9983 |
| Nanyang | 120.19933 | 212.62944 | 332.82876 | 16.47074 |
| Mohe | 576.95191 | 116.3 | 693.25191 | 12.35433 |
| Yichang | 91.65438 | 207.28921 | 298.9436 | 16.16286 |
| Shaoyang | 88.53056 | 211.08337 | 299.61393 | 15.43056 |
| Xuzhou | 137.82225 | 206.70764 | 344.52989 | 16.0768 |
| Jingdezhen | 81.65978 | 238.09146 | 319.75124 | 15.72363 |
| Dalian | 232.43348 | 158.17483 | 390.60831 | 16.2524 |
| Chifeng | 321.75303 | 164.21303 | 485.96607 | 15.75758 |
| Yanchi | 231.83865 | 181.03191 | 412.87056 | 17.20097 |
| Yushu | 305.97348 | 132.56022 | 438.53371 | 20.09532 |
| Qingdao | 174.1991 | 182.91663 | 357.11573 | 16.23196 |
| Datong | 294.12966 | 165.18831 | 459.31798 | 15.92942 |
| Xianyang | 144.36854 | 206.3382 | 350.70674 | 17.17031 |
| Shanghai | 95.76157 | 200.14449 | 295.90607 | 15.75319 |
| Daocheng | 273.11865 | 99.19798 | 372.31663 | 20.47532 |
| Taipei | 11.53506 | 263.12989 | 274.66494 | 12.79728 |
| Tianjin | 202.67933 | 199.87371 | 402.55303 | 15.66885 |
| Nagqu | 357.10202 | 90.37573 | 447.47775 | 18.59243 |
| Hong Kong | 5.33146 | 306.62404 | 311.95551 | 13.34291 |
| Kashi | 199.36742 | 221.1773 | 420.54472 | 16.81435 |
| Lijiang | 111.98831 | 209.46517 | 321.45348 | 22.18575 |
| Wenzhou | 62.69191 | 228.49461 | 291.18652 | 15.64445 |
| Chongqing | 79.02966 | 177.24157 | 256.27124 | 15.24826 |

**Supplementary Table S7**

Cost-benefit analysis of ESW-PZ vs Low-E glass.

|  | Low-E glass | ESW-PZ |
| --- | --- | --- |
| Window Opening Area (m^2^) | 35851.3 | 35851.3 |
| production cost (Yuan/m^2^) | 240 | 360 |
| total price (Yuan) | 8,604,312 | 12,906,468 |
| average energy saving | / | 13121.6 GJ (312 tonnes of petroleum) |
| Total oil price (Yuan) | / | 1,201,824 |
| payback time (Year) | / | 3.6 |

**Supplementary Table S8**

Performance comparison of ESW-PZ with other similar electrochromic energy storage devices.

| Material | Voltage window | Optical contrast | Coloration efficiency | Capacitance | Power density | Energy density | Refs. |
| --- | --- | --- | --- | --- | --- | --- | --- |
| Mo-doped WO_3_// MnO_2_ | 0-2.0 V | >60%, 420-800 nm | 84.3 cm^2^ C^−1^ | 19.1 mF cm^-2^ | 0.112 mW cm^-2^ | 11 µWh cm^-2^ | [S1] |
| Li_4_Ti_5_O_12_//LiMn_2_O_4_ | 0-3.0 V | 35.2%, 850 nm |  |  | 0.22 mW cm^-2^ | 14 µWh cm^-2^ | [S2] |
| PANI//WO_3_ | 0-1.4 V | 43% , 633 nm |  | 28.3 mF cm^-2^ | 0.13 mW cm^−2^ | 7.7 µWh cm^-2^ | [S3] |
| V_2_O_5_//ZPEM | -0.5-2.0 V | 57.88%, 700 nm | 36.91 cm^2^ C^−1^ | 12.29 mF cm^−2^ | 1.67 mW cm^−2^ | 12.54 μWh cm^−2^ | [S4] |
| W_18_O_49_ NW/rGO composite | 0-1.1 V |  | 46 cm^2^ C ^-1^ | 48 mF cm^-2^ | 0.391 mW cm^-2^ | 5.2 μW h cm^-2^ | [S5] |
| PETC//V_2_O_5_ | 0-2.0 V | 67%, 1062 nm | 132 cm^2^ C^−1^ | 32.9 mF cm^-2^ | 2.5 mW cm^-2^ | 18.2 µWh cm^-2^ | [S6] |
| Ta-doped nano-TiO_2_/ NiO | -3.5-1 V | 89.1%, 550 nm | 121.2 cm^2^ C^−1^ | 466.5 mA hr m^2^ |  |  | [S7] |
| P5ICN/TiO_2_/PEDOT | 0-1.5 V | 54%, 600 nm | 648 cm^2^ C^−1^ | 14.7 mF cm^-2^ |  |  | [S8] |
| **EPADO//Zn** | **0-2.6 V** | **67.13%, 750 nm**  **68.06%, 1250 nm** | **61 cm^2^ C^−1^**  **57 cm^2^ C^−1^** | **135.85 mF cm^-2^** | **3.12 mW cm^-2^** | **127.54 µWh cm^-2^** | **This work** |

**S-references**

[S1] S. Xie, Y. Chen, Z. Bi, S. Jia, X. Guo, X. Gao, X. Li, *Chem. Eng. J.* **2019**, *370*, 1459.

[S2] W. Li, T. Bai, Q. Zhang, J. Liu, K. Zhou, H. Wang, *J. Mater. Chem. C* **2023**, *11*, 7740.

[S3] Y. Zhong, Z. Chai, Z. Liang, P. Sun, W. Xie, C. Zhao, W. Mai, *ACS Appl. Mater. Interfaces* **2017**, *9*, 34085.

[S4] C. Wang, X. Zhang, S. Liu, H. Zhang, Q. Wang, C. Zhang, J. Gao, L. Liang, H. Cao, *ACS Appl. Energy Mater.* **2022**, *5*, 88.

[S5] M. Hassan, G. Abbas, Y. Lu, Z. Wang, Z. Peng, *J. Mater. Chem. A* **2022**, *10*, 4870.

[S6] Y. Zhang, F.-Q. Bai, Y. Xie, M. Zhu, L. Zhao, D. An, D. Xue, E. B. Berda, C. Wang, G. Lu, X. Jia, D. Chao, *Chem. Eng. J.* **2022**, *450*, 138386.

[S7] S. Cao, S. Zhang, T. Zhang, Q. Yao, J. Y. Lee, *Joule* **2019,** *3,* 1152.

[S8] Z. Li, B. Wang, Y. Tian, X. Zhao, Q. Guo, G. Nie, *Synth. Met.* **2021,** *277,* 116785.
